# Supplementary material for: Feature-Assisted Machine Learning for Predicting Band Gaps of Binary Semiconductors
Source: Nanomaterials (Basel). 2024 Feb 28;14(5):445. doi: 10.3390/nano14050445 (PMC10935296; doi:10.3390/nano14050445)
Supplement: Supplementary file 1 [file nanomaterials-14-00445-s001.zip › nanomaterials-2876995-supplementary.pdf]

Supporting Information for

Feature-Assisted Machine Learning for Predicting Band Gaps of Binary Semiconductors

Sitong Huo, Shuqing Zhang *, Qilin Wu and Xinping Zhang *

Institute of Information Photonics Technology, School of Physics and Optoelectronic Engineering, Beijing University of Technology, Beijing 100124, China; hst6110@emails.bjut.edu.cn (S.H.); qlwu2001@emails.bjut.edu.cn (Q.W.)
* Correspondence: zhangsq@bjut.edu.cn (S.Z.); zhangxinping@bjut.edu.cn (X.Z.)

In this work, we utilized six supervised learning algorithms, LASSO, Kernel Ridge Regression (KRR), Support Vector regression (SVR), Decision Trees (DT), Random Forests (RF), and GBDT. All these models are implemented in python 3.8 environment and training and optimization is done based on Scikit-learn[1] framework. Our machine learning models employed three-fold cross-validation. The specific model parameters are given in Table S1.

Table S1. Parameters of traditional machine learning models.

Model	Main parameters
Lasso	selection = 'cyclic', alpha =0.04, max_iter = 1200
Kernel Ridge	kernel = 'rbf'
Support Vector Regression	kernel = 'rbf', gamma = 'scale'
Decision Tree	Splitter = "best", criterion = "mse"
Random Forest	max_depth = 30
Gradient Boosting Regressor	n_estimators = 358, learning_rate = 0.1

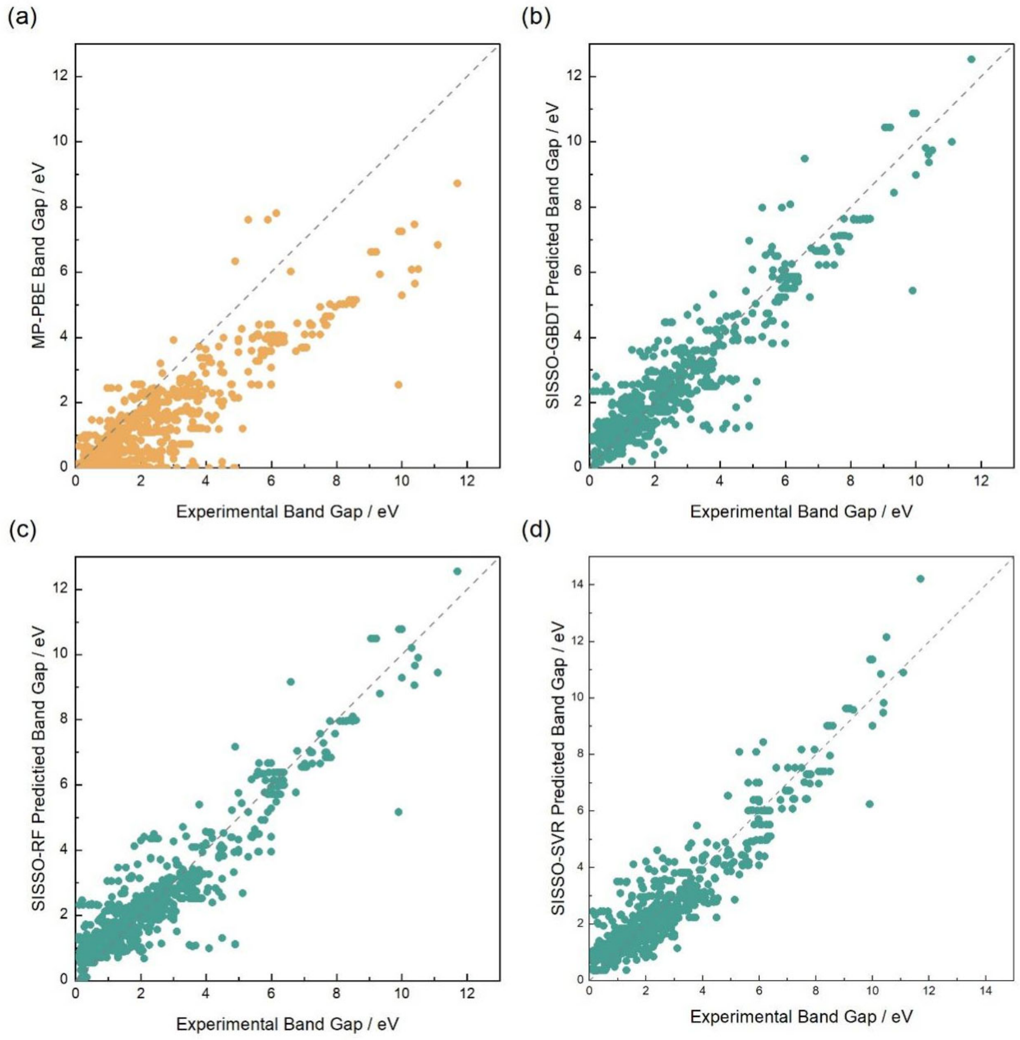

Figure S1. (**a**) Comparison of experimental band gap values with PBE-calculated band gaps from the Materials Project database. (**b**–**d**) Comparison of band gap values predicted by three stable SISSO 2D models. Using the top 11 from SVR as inputs for the SISSO-SVR method (**b**), the top 7 from RF as inputs for the SISSO-RF method (**c**), and the top 4 features from GBDT as inputs for the SISSO-GBDT method (**d**).

Table S2. Predicted band gap values for all stable binary semiconductors in the Materials Project database. The band gap values from left to right are the PBE calculated values and the predicted band gap values of the stable SISSO model based on GBDT, RF and SVR respectively.

Unit: eV

Formula \ E_g	PBE	SISSO-GBDT	SISSO-RF	SISSO-SVR	Formula \ E_g	PBE	SISSO-GBDT	SISSO-RF	SISSO-SVR
Ac_2O_3	3.56	6.49	6.31	6.37	As_2O_5	1.43	2.74	2.94	3.50
Ac_2S_3	2.27	4.15	3.42	4.07	As_2Os	0.76	0.56	0.95	0.07
$AcBr_3$	4.11	6.40	6.38	6.89	As_2Pt	0.29	0.30	0.54	0
$AcCl_3$	5.11	7.62	7.64	8.15	As_2Rh	0.31	0.32	0.56	0
AcF_3	6.21	9.66	9.64	11.21	As_2Ru	0.56	0.40	0.74	0
AcH_3	0.68	2.09	2.00	3.93	As_2S_3	2.20	2.26	2.82	2.45
AcI_3	2.59	4.56	4.33	5.18	As_2Se_3	0.63	0.97	1.18	1.48
$AcMg_{149}$	0.22	0.11	0.58	0.58	As_3Ir	0.03	0	0.18	0
AcN	0.06	2.65	2.23	4.04	$AsBr_3$	3.20	3.57	4.30	4.33
Ag_2O	0.06	2.04	1.78	1.71	$AsCl_3$	4.10	4.54	5.45	5.24
Ag_2S	1.39	2.04	2.27	1.58	$AsCl_5$	1.46	2.42	2.68	4.36
Ag_3P_{11}	1.10	1.24	1.55	0.95	AsF_3	5.40	6.52	7.67	7.61
AgBr	1.17	2.37	2.44	2.49	AsF_5	4.63	5.91	6.86	7.67
AgCl	1.20	2.64	2.68	2.87	AsI_3	2.25	2.41	2.84	3.32
AgF_3	0.21	2.78	2.50	3.83	AsS	2.12	2.20	2.77	2.97
AgI	1.72	2.43	2.70	2.40	AsS_2	1.29	1.54	1.90	2.37
Al_2O_3	6.04	7.59	8.37	5.35	Au_2O_3	0.89	1.84	2.01	1.33
Al_2Os	0.31	1.37	1.08	0	Au_2S	1.91	1.53	2.17	1.17
Al2Ru	0.10	1.20	0.85	0	Au_3F_8	0.55	2.20	2.22	5.78
Al_2S_3	3.24	4.27	4.54	2.94	AuBr	2.06	2.19	2.73	2.16
Al_2Se_3	2.16	3.35	2.55	2.22	$AuBr_3$	0.81	1.15	1.42	1.64
Al_2Te_3	1.84	2.46	2.09	1.82	AuCl	2.08	2.47	2.96	2.44
Al_2Te_5	1.13	1.89	1.82	1.71	$AuCl_2$	0.96	1.55	1.79	1.85
Al_4C_3	1.34	2.69	2.52	1.39	$AuCl_3$	1.39	1.90	2.24	2.13
AlAs	1.69	2.46	2.33	2.12	AuF_3	1.55	3.03	3.27	3.25
$AlBr_3$	4.10	5.45	5.84	4.67	AuF_5	0.79	2.40	2.47	3.53
$AlCl_3$	5.31	6.67	7.31	5.71	AuI	2.09	1.80	2.45	1.85
AlF_3	7.72	9.55	10.70	8.60	AuSe	0.32	0.17	0.48	0.61
AlH_3	3.25	3.76	4.15	9.84	B_2H_5	6.35	5.33	6.94	8.10
AlI_3	3.27	4.40	4.65	3.74	B_2O_3	6.34	6.96	8.23	6.53
AlN	4.05	5.51	5.87	4.98	B_2S_3	2.53	2.78	3.23	3.21
AlP	1.82	2.58	2.20	2.40	B_5H_7	3.56	3.07	4.02	2.14
AlSb	1.26	1.92	1.91	1.50	B_6As	2.97	2.56	3.39	2.94
As_2Ir	1.15	0.88	1.36	0.38	B_6O	2.21	3.61	3.56	4.34
As_2O_3	3.63	4.50	5.25	4.25	B_6P	2.73	2.37	3.14	3.02
B_9H_{11}	2.91	2.54	3.35	0.29	$BeBr_2$	5.35	6.84	7.18	7.25

Ba_2Ge	0.05	1.27	1.36	0.40	$BeCl_2$	6.34	8.11	8.44	8.48
Ba_2Pb	0.04	1.73	1.68	0.25	BeF_2	7.96	10.80	10.99	11.75
Ba_2Sb_3	0.27	1.55	1.63	1.11	BeH_2	5.40	5.75	6.44	16.70
Ba_2Si	0.06	1.10	1.25	0.48	BeI_2	4.08	5.14	5.54	5.74
Ba_2Sn	0.02	1.15	1.27	0.22	BeO	7.46	9.60	9.90	9.47
Ba_3As_{14}	1.18	2.65	2.12	1.68	BeP_2	0.86	1.20	1.68	2.69
Ba_3P_{14}	1.92	3.40	2.93	2.19	BeS	3.24	4.18	4.58	4.89
Ba_3P_2	0.22	1.71	1.73	1.63	BeSe	2.90	3.80	4.19	4.37
Ba_3P_4	0.48	1.97	2.00	1.82	BeTe	2.21	2.40	3.00	3.53
Ba_3Si_4	0.00	1.05	1.19	0.50	BF_3	8.16	9.05	10.70	10.45
Ba_4P_3	0.75	2.23	2.28	1.87	BH_3	5.28	4.47	5.83	14.66
Ba_5P_4	0.96	2.45	2.35	1.98	Bi_2O_3	2.25	3.67	3.49	3.02
Ba_5Sb_4	0.27	1.55	1.63	1.00	Bi_2S_3	1.50	1.99	2.29	1.72
$BaAs_2$	0.43	1.90	1.94	1.74	Bi_2Se_3	0.54	1.19	1.26	1.12
BaB_6	0.06	1.32	1.40	3.95	Bi_2Te_3	0.39	0.41	0.62	0.83
$BaBr_2$	4.37	6.92	6.88	5.83	BI_3	0.00	0.84	0.78	2.67
$BaCl_2$	5.61	8.41	8.38	7.08	Bi_3Sb	0.08	0.08	0.24	0.13
BaF_2	6.62	10.43	10.30	9.62	Bi_4O_7	0.90	2.59	2.56	3.23
BaF_3	0.88	4.69	4.29	6.19	Bi_4Te_3	0.26	0.30	0.49	0.55
$BaGe_2$	0.57	1.79	1.91	0.89	$BiBr_3$	2.91	3.61	4.05	3.58
BaH_2	2.87	4.37	4.51	14.49	$BiCl_3$	3.43	4.27	4.91	4.21
BaI_2	3.44	5.58	5.58	5.05	BiF_3	4.70	6.23	7.10	6.30
$BaMg_{149}$	0.36	0.36	0.95	0.12	BiF_5	1.90	4.00	4.18	5.44
BaN_6	3.81	6.46	6.38	6.26	BiI_3	2.56	2.95	3.27	2.96
BaO	2.26	5.41	2.69	4.89	BiO_2	1.08	2.73	2.75	2.84
BaO_2	2.29	5.45	2.79	4.67	BiS_2	1.19	1.74	1.96	1.92
BaP_2	0.90	2.39	2.42	2.23	BN	4.49	4.98	5.88	6.18
BaP_3	2.12	2.20	2.77	2.97	BP	1.47	1.35	1.83	2.73
BaS	1.29	1.54	1.90	2.37	Br_2O_3	1.79	2.02	2.51	2.72
BaS_2	0.89	1.84	2.01	1.33	BrCl	1.83	1.70	2.26	3.47
BaS_3	1.91	1.53	2.17	1.17	BrF_3	2.34	3.09	3.65	5.21
BaSe	0.55	2.20	2.22	5.78	BrF_5	3.46	3.97	3.78	6.37
$BaSe_2$	2.06	2.19	2.73	2.16	BS_2	2.55	2.80	3.32	3.61
$BaSi_2$	0.81	1.15	1.42	1.64	BSe_2	1.77	2.12	2.52	2.92
$BaSn_2$	2.08	2.47	2.96	2.44	Ca_2As_3	0.17	1.42	1.54	1.85
BaTe	0.96	1.55	1.79	1.85	Ca_2Bi	0.05	1.06	1.26	0.76
BBr_3	1.39	1.90	2.24	2.13	Ca_2Ge	0.31	1.30	1.52	0.84
BCl_3	1.55	3.03	3.27	3.25	Ca_2Pb	0.08	1.55	1.61	0.52
Be_2C	0.79	2.40	2.47	3.53	Ca_2Si	0.29	1.10	1.38	0.92
Be_3N_2	3.72	5.32	5.56	5.49	Ca_2Sn	0.06	0.97	1.20	0.48
Be_3P_2	0.89	1.23	1.71	2.29	$Ca_{31}Sn_{20}$	0.04	0.94	1.18	0.28
Be_5Pt	0.15	0.64	1.04	0	Ca_3N_2	1.11	3.56	3.20	4.11
Ca_4As_3	0.69	1.94	2.09	2.01	CO_2	6.64	6.42	8.01	7.60
Ca_5P_8	1.19	2.46	2.22	3.75	$CoAs_2$	0.17	0.50	0.63	1.05

Ca_5Sb_3	0.25	1.30	1.50	1.29	$CoBr_2$	0.12	1.55	1.39	2.20
CaB_6	3.72	5.32	5.56	5.49	$CoCl_2$	0.10	1.78	1.58	2.58
$CaBr_2$	0.89	1.23	1.71	2.29	CoF_2	2.27	4.59	3.89	5.19
$CaCl_2$	0.15	0.64	1.04	0	CoF_3	2.05	4.40	4.12	5.13
CaF_2	5.35	6.84	7.18	7.25	CoO	0.84	2.75	2.65	2.77
CaH_2	6.34	8.11	8.44	8.48	CoO_2	0.59	2.54	2.38	2.63
CaI_2	7.96	10.80	10.99	11.75	CoP_2	0.54	0.83	1.03	1.41
$CaMg_{149}$	5.40	5.75	6.44	16.70	$CoSb_3$	0.58	0.64	0.92	1.07
CaN_6	4.08	5.14	5.54	5.74	Cr_2O_3	2.43	4.49	2.92	3.24
CaO	7.46	9.60	9.90	9.47	Cr_5O_{12}	1.82	3.95	3.56	2.60
CaP	0.86	1.20	1.68	2.69	$CrBr_3$	1.29	2.88	2.84	2.90
CaP_3	3.24	4.18	4.58	4.89	$CrCl_3$	0.18	2.15	1.89	2.77
CaS	2.90	3.80	4.19	4.37	CrF_3	3.09	5.70	3.92	5.68
CaSe	2.14	3.92	3.13	3.87	CrF_4	1.57	4.36	4.20	5.09
CaTe	1.60	2.73	1.93	3.15	CrF_5	1.46	4.26	4.09	5.27
CCl_4	4.51	4.36	5.50	6.89	CrF_6	2.09	4.82	3.85	6.02
$CdAs_2$	0.19	1.02	0.85	1.28	CrI_3	1.16	2.36	2.40	2.37
$CdBr_2$	2.94	4.31	4.50	4.13	CS_{14}	2.70	2.12	2.99	3.48
$CdCl_2$	3.59	5.09	5.43	4.92	Cs_2As_3	0.68	1.57	2.30	1.58
CdF_2	3.07	5.60	3.84	6.32	Cs_2In_3	2.14	3.92	3.13	3.87
CdI_2	2.44	3.52	3.24	3.34	Cs_2O	1.60	2.73	1.93	3.15
CdP_2	1.59	2.18	2.32	2.18	Cs_2O_2	4.51	4.36	5.50	6.89
CdS	1.12	2.33	2.23	2.28	Cs_2P_3	0.19	1.02	0.85	1.28
CdS_2	1.54	2.68	2.67	2.44	Cs_2S	2.94	4.31	4.50	4.13
CdSb	0.05	0.71	0.57	0.77	Cs_2S_3	3.59	5.09	5.43	4.92
CdSe	0.51	1.80	1.57	1.69	Cs_2S_5	3.07	5.60	3.84	6.32
CdTe	1.23	1.75	1.85	1.90	Cs_2Sb	2.44	3.52	3.24	3.34
Ce_2Br_5	0.00	1.62	2.06	2.32	Cs_2Se	1.59	2.18	2.32	2.18
$CeAs_2$	0.07	1.40	1.26	1.04	Cs_2Se_3	1.12	2.33	2.23	2.28
$CeBr_3$	0.01	1.62	2.06	2.42	Cs_2Se_5	1.54	2.68	2.67	2.44
$CeCl_3$	0.01	1.71	2.27	2.84	Cs_2Te	0.05	0.71	0.57	0.77
CeF_4	2.15	3.80	1.45	5.56	Cs_2Te_3	0.55	1.30	2.08	1.62
CeO_2	1.88	3.30	1.39	3.45	Cs_3As_{11}	1.39	2.33	1.79	1.75
CF_4	9.07	9.00	11.12	11.65	Cs_3As_7	1.57	2.53	1.96	3.68
Cl_2O	1.15	1.32	1.63	2.62	Cs_3Bi	0.36	0.97	1.80	0.57
Cl_2O_7	3.68	3.31	4.27	7.14	Cs_3P_7	2.34	3.38	4.05	4.64
ClF	2.09	2.71	3.18	5.45	Cs_3Sb	1.10	1.82	1.95	1.49
ClF_3	2.54	3.06	3.65	5.67	Cs_4Pb_9	0.82	1.95	2.54	0.56
ClF_5	3.12	3.52	4.26	6.64	Cs_5Sb_8	0.58	1.26	2.06	1.86
ClO_2	0.97	1.18	1.44	3.04	CsAu	1.99	3.51	2.88	1.29
ClO_3	1.21	1.37	1.69	3.34	CsBi	0.25	0.85	1.68	0.67
CsBr	4.42	6.73	7.04	6.08	$ErCl_3$	2.34	3.38	4.05	4.64
$CsBr_3$	2.32	4.46	3.51	4.31	ErF_3	1.10	1.82	1.95	1.49
CsCl	5.02	7.64	7.87	6.98	ErH_3	0.82	1.95	2.54	0.56

CsF	5.28	8.98	9.00	9.02	ErI_3	0.58	1.26	2.06	1.86
$CsGa_3$	0.13	0.38	1.33	0	$ErMg_{149}$	1.99	3.51	2.88	1.29
$CsGe$	1.20	1.87	1.80	1.21	ErN	0.25	0.85	1.68	0.67
CsH	2.59	3.66	4.32	12.82	ErS_2	4.42	6.73	7.04	6.08
CsI	3.86	5.70	6.13	5.11	$EuBr_2$	2.32	4.46	3.51	4.31
CsI_3	2.02	3.71	2.85	3.58	$EuCl_2$	1.52	2.87	2.12	3.41
CsI_4	1.78	3.46	2.13	3.50	EuI_2	1.23	2.46	2.08	2.39
$CsIn_3$	0.17	0.37	1.35	0	$EuMg_{149}$	0.02	1.45	0.26	0.77
CsN_3	4.26	6.66	6.95	5.04	EuP_7	0.39	1.69	1.32	6.21
CsP_7	1.28	2.23	1.92	5.91	$FeAs_2$	0.29	0.66	0.80	1.16
CsS	1.73	3.29	2.05	3.09	$FeBr_3$	0.03	1.53	1.35	2.36
CsS_3	1.68	3.23	1.90	2.87	$FeCl_3$	0.43	2.13	1.98	2.95
$CsSb$	0.56	1.23	2.03	1.30	$FeCl_4$	0.28	2.00	1.82	3.04
$CsSe_4$	0.97	2.43	2.61	2.36	FeF_2	1.74	4.23	4.21	5.09
$CsSi$	1.56	2.08	2.23	1.51	FeF_3	3.07	5.36	3.68	5.77
$CsSn$	1.14	1.71	1.82	1.01	FeO	2.30	4.08	3.24	3.77
$CsTe$	1.01	1.79	2.11	2.10	FeP_2	0.47	0.83	1.00	1.43
$CsTe_4$	0.63	1.39	2.16	1.91	FeP_4	0.90	1.20	1.45	2.01
Cu_2O	0.51	2.46	2.28	2.46	FeS_2	0.98	1.84	1.94	1.92
Cu_2P_7	0.91	1.14	1.39	5.47	$FeSe_2$	0.40	1.30	1.31	1.45
$CuBr$	0.49	1.86	1.75	2.54	$FeSi$	0.17	0.10	0.38	0.40
CuI	1.46	2.27	2.46	2.69	$FeSi_2$	0.70	0.56	0.94	0.75
CuP_{10}	1.04	1.25	1.53	2.55	$FeTe_2$	0.12	0.39	0.55	1.08
CuP_2	0.96	1.19	1.45	1.73	Ga_2I_3	2.25	3.12	2.53	2.42
Dy_2O_3	3.94	4.72	4.29	4.12	Ga_2O_3	2.04	3.90	3.49	2.91
Dy_2S_3	0.59	1.88	1.83	1.56	Ga_2Ru	0.12	0.75	0.67	0
Dy_2Se_3	0.57	1.86	1.79	1.36	Ga_2S_3	2.03	2.83	2.61	1.95
Dy_2Te_3	0.79	1.92	1.42	1.22	Ga_2Se_3	1.68	2.51	2.67	1.62
Dy_6Se_{11}	0.53	1.83	1.77	1.18	Ga_2Te_3	1.01	1.32	1.50	1.10
$DyBr_3$	3.01	3.83	3.36	3.86	Ga_2Te_5	1.02	1.32	1.50	1.37
$DyCl_3$	3.90	4.57	3.98	4.70	Ga_3Fe	0.58	0.53	0.76	0
DyF_3	7.87	7.93	6.68	8.22	Ga_3Os	0.97	1.43	1.55	0
DyH_3	0.01	1.35	1.16	3.46	Ga_3Ru	0.72	1.23	1.30	0
DyI_3	2.18	3.09	1.65	2.97	Ga_7Te_{10}	0.90	1.23	1.38	0.91
$DyMg_{149}$	0.26	1.59	0.35	0.82	$GaAs$	0.18	0.77	0.71	0.92
DyN	0.21	1.76	2.14	2.36	$GaBr_2$	3.11	4.19	4.59	3.63
Er_2O_3	0.55	1.30	2.08	1.62	$GaBr_3$	3.36	4.40	4.86	3.75
Er_2S_3	1.39	2.33	1.79	1.75	$GaCl_2$	4.03	5.19	5.76	4.47
Er_2Se_3	1.57	2.53	1.96	3.68	$GaCl_3$	4.28	5.39	6.02	4.56
Er_2Te_3	0.36	0.97	1.80	0.57	GaF_3	4.84	6.78	7.47	6.30
GaI_2	2.38	3.22	2.94	2.81	H_3N	4.34	5.63	5.56	7.94
GaI_3	2.39	3.22	2.95	2.84	H_3S	4.35	4.90	5.08	6.63
GaN	1.74	3.18	3.24	3.07	H_4C	7.64	8.40	8.49	8.17
GaP	1.68	2.00	2.29	1.99	HBr	4.94	6.16	6.10	9.30

GaS	2.01	2.81	2.62	2.44	HCl	5.97	7.57	7.39	10.95
GaSb	0.37	0.71	0.77	0.72	HF	6.72	9.56	9.03	14.42
GaSe	1.26	2.16	2.22	1.80	Hf_3N_4	1.01	3.29	3.01	2.77
GaTe	0.90	1.22	1.38	1.37	$HfBr_4$	4.29	6.23	6.36	4.90
$Gd_{10}S_{19}$	0.34	1.71	1.72	1.22	$HfCl_4$	4.18	6.39	6.46	5.28
Gd_2C_{l3}	0.09	1.74	2.22	2.68	HfF_4	5.46	8.55	8.65	7.61
Gd_2O_3	2.94	4.02	2.67	3.75	HfI_4	2.31	4.00	3.25	3.39
Gd_2S_3	0.46	1.79	1.77	1.55	$HfMg_{149}$	0.14	0	0.29	1.00
Gd_2Se_3	0.62	1.90	1.81	1.43	HfO_2	4.07	6.62	6.63	4.85
Gd_2Te_3	0.10	1.40	1.12	0.95	HfS_2	1.23	2.89	2.76	2.29
$GdBr_3$	1.52	2.87	2.12	3.41	HfS_3	1.14	2.81	2.66	2.29
$GdCl_3$	1.23	2.46	2.08	2.39	$HfSe_2$	0.55	2.23	2.02	1.74
GdF_3	0.02	1.45	0.26	0.77	$HfSe_3$	0.61	2.28	2.08	1.84
GdH_3	0.39	1.69	1.32	6.21	HgBr	2.46	3.31	2.75	3.13
GdP_5	0.29	0.66	0.80	1.16	$HgBr_2$	2.84	3.61	3.86	3.09
GdS_2	0.03	1.53	1.35	2.36	HgCl	2.82	3.85	3.59	3.69
Ge_3F_8	0.43	2.13	1.98	2.95	$HgCl_2$	3.17	4.14	4.67	3.60
Ge_3N_4	0.28	2.00	1.82	3.04	HgF	1.98	4.11	4.28	4.53
Ge_3Ru_2	1.74	4.23	4.21	5.09	HgF_2	1.17	3.45	3.43	4.00
Ge_5F_{12}	3.07	5.36	3.68	5.77	HgI	1.89	2.44	2.80	2.38
Ge_5Ir_4	2.30	4.08	3.24	3.77	HgI_2	1.46	2.09	2.35	2.04
$GeAs_2$	0.47	0.83	1.00	1.43	HgO	1.25	2.90	2.95	2.58
$GeBr_2$	2.60	3.39	3.09	3.71	HgS_4	1.05	1.64	1.84	1.88
$GeBr_4$	3.08	3.77	4.35	4.11	HgSe	0.12	0.85	0.84	0.90
$GeCl_4$	4.27	4.98	5.80	5.10	HI	4.08	4.75	4.89	7.66
GeF_2	4.03	5.73	6.42	6.48	$Ho_{13}Zn_{58}$	0.05	1.35	0.60	0
GeF_4	5.68	7.06	8.14	7.39	Ho_2O_3	3.96	4.73	4.37	4.09
GeH_4	5.78	4.91	6.39	13.16	Ho_2S_3	1.46	2.53	1.77	1.93
GeI_2	2.07	2.58	2.85	2.94	Ho_2Se_3	2.60	3.39	3.09	3.71
GeI_4	2.00	2.51	2.90	3.11	Ho_2Te_3	3.08	3.77	4.35	4.11
GeO_2	3.26	4.51	4.68	4.20	Ho_4C_7	4.27	4.98	5.80	5.10
GeS	1.24	1.79	2.02	2.20	$HoBr_3$	4.03	5.73	6.42	6.48
GeS_2	2.26	2.62	2.58	2.66	$HoCl_3$	5.68	7.06	8.14	7.39
GeSe	0.44	1.11	1.16	1.51	HoF_3	5.78	4.91	6.39	13.16
$GeSe_2$	1.66	2.10	2.44	2.17	HoH_3	2.07	2.58	2.85	2.94
GeTe	0.82	0.76	1.09	1.51	HoI_3	2.00	2.51	2.90	3.11
H_2O	5.53	7.51	7.22	9.57	$HoMg_{149}$	3.26	4.51	4.68	4.20
H_2S	4.37	4.93	5.11	6.76	HoN	1.24	1.79	2.02	2.20
H_3F_2	7.91	10.84	10.27	14.15	I_2O_5	2.26	2.62	2.58	2.66
IBr	0.44	1.11	1.16	1.51	K_2S_5	1.95	3.49	2.69	3.53
ICl	1.86	2.05	2.61	3.27	K_2Se	2.09	3.60	3.18	3.53
ICl_3	1.81	2.02	2.56	3.23	K_2Se_3	0.65	2.05	2.63	2.42
IF_5	4.68	5.25	6.41	6.47	K_2Se_5	0.94	2.36	2.68	2.70
IF_7	1.55	2.78	3.14	7.43	K_2Te	2.14	2.98	3.71	3.25

In_2Br_3	2.13	3.46	2.87	2.48	K_2Te_3	0.68	1.41	2.19	2.13
In_2O_3	0.93	3.07	2.85	2.11	K_3As	0.11	0.92	1.67	1.61
In_2S_3	0.83	1.92	1.84	1.19	K_3As_{11}	1.47	2.38	1.73	2.23
In_2Se_3	0.99	2.01	1.98	1.09	K_3As_7	1.40	2.31	1.81	4.01
In_2Te_5	0.85	1.25	1.36	1.09	K_3Bi	0.03	0.57	1.42	0.63
In_3Ru	0.25	0.91	0.83	0	K_3Bi	0.04	0.58	1.43	0.64
In_4Te_3	0.20	0.72	0.68	0.37	K_3Ga_{13}	0.06	0.27	1.23	0
In_7Cl_9	2.19	3.76	3.01	4.38	K_3P_{11}	1.93	2.90	3.05	2.69
In_7Te_{10}	0.81	1.22	1.32	0.68	K_3Sb	0.39	1.01	1.83	1.37
$InAs$	0.31	0.93	0.87	0.81	$K_{49}Tl_{108}$	0.05	0.63	1.46	0
$InBr$	1.62	3.05	3.06	2.67	K_4Sn_9	1.12	1.66	1.87	0.95
$InBr_2$	2.42	3.70	2.77	2.93	KAs	1.13	2.01	2.10	2.50
$InBr_3$	2.76	3.97	3.84	3.10	KBi	0.41	0.98	1.82	1.08
$InCl$	2.19	3.75	3.01	3.36	KBr	4.52	6.79	7.10	7.01
$InCl_2$	3.02	4.43	4.46	3.57	KCl	5.04	7.64	7.86	7.96
$InCl_3$	3.03	4.44	4.48	3.57	KF	6.07	9.81	9.79	10.85
InF_3	4.03	6.18	6.65	5.37	KF_2	1.47	4.85	3.61	7.20
InI	1.74	2.77	2.88	2.33	KGa_3	0.62	0.87	1.82	0.38
InI_2	1.79	2.80	2.91	2.22	KGe	1.33	1.96	1.71	1.61
InI_3	2.34	3.25	2.83	2.69	KH	3.54	4.64	5.28	17.07
InN	0.48	2.22	1.95	2.03	KI	4.04	5.87	6.29	6.01
InP	0.47	1.08	1.05	1.07	KN_3	4.20	6.56	6.85	5.80
InS	1.55	2.50	2.59	1.90	KP	1.48	2.41	1.73	3.01
$InSe$	0.66	1.74	1.63	1.22	KP_{15}	1.52	2.45	1.77	2.52
Ir_2S_3	1.15	1.38	1.73	0.92	KPb	0.37	1.43	2.11	0.80
$IrBr_3$	1.63	2.31	2.64	2.14	KrF_2	2.85	6.34	4.07	8.08
$IrCl_3$	1.94	2.83	3.17	2.56	KS	1.67	3.19	1.95	3.60
IrF_3	1.17	3.17	3.22	3.42	KS_3	1.80	3.33	2.24	3.50
IrF_6	0.78	2.84	2.82	4.32	KSb	0.88	1.54	2.22	1.90
IrI_3	1.40	1.70	2.08	1.70	KSb_2	0.27	0.89	1.71	1.48
IrS_2	0.56	0.89	1.12	1.04	KSe	0.98	2.40	2.64	2.84
$IrSe_2$	0.07	0.42	0.57	0.70	KSi	1.26	1.71	1.66	1.65
K_2Ga_3	0.28	0.50	1.46	0	KSn	0.73	1.24	2.09	1.03
K_2O	1.72	4.42	2.79	4.88	KTe	0.67	1.40	2.18	2.31
K_2O_2	2.40	5.15	3.20	5.08	$La_{10}S_{19}$	0.72	2.67	2.44	1.41
K_2P_3	1.09	1.99	2.14	2.47	$La_{10}Se_{19}$	0.57	2.48	2.25	1.16
K_2S	2.32	3.89	3.86	3.95	La_2I_5	0.18	2.26	1.95	1.98
K_2S_3	1.32	2.81	2.31	3.05	La_2O_3	3.59	6.51	6.34	4.11
La_2S_3	1.04	2.97	2.77	1.86	Lu_2Te_3	0.59	1.78	1.33	1.24
La_2Se_3	1.54	3.41	2.34	1.90	$LuBr_3$	3.10	3.85	3.62	4.11
La_2Te_3	1.00	2.24	2.23	1.41	$LuCl_3$	3.85	4.48	4.25	4.92
La_4Pb_3	0.05	1.68	1.48	0	LuF_3	7.69	7.67	7.25	8.51
La_7Ru_3	0.04	1.48	1.33	0	LuH_3	0.11	1.44	1.17	3.82
$LaAs$	0.04	1.45	1.31	1.00	LuI_3	2.18	3.07	1.80	3.15

$LaBr_2$	0.59	3.05	2.70	2.73	$LuMg_{149}$	0.33	1.63	0.36	0.93
$LaBr_3$	2.93	5.28	5.07	3.94	LuN	0.38	1.88	2.20	2.64
$LaCl_3$	3.67	6.24	6.13	4.73	$Mg_{149}Ag$	0.57	0.86	1.38	0
LaF_3	6.02	9.49	9.45	7.53	$Mg_{149}Al$	0.50	0.22	0.97	0
LaI_3	2.07	4.07	2.70	3.02	$Mg_{149}Au$	0.56	1.81	2.01	0.14
LaN	1.15	3.68	3.26	3.04	$Mg_{149}Be$	0.53	0.17	0.96	0.52
LaP_2	0.63	2.03	1.93	1.55	$Mg_{149}Bi$	0.29	0.74	1.18	0.62
LaP_5	0.11	1.53	1.39	1.82	$Mg_{149}Br$	0.00	1.85	1.86	3.26
LaP_7	0.87	2.26	2.19	6.37	$Mg_{149}Cd$	0.57	0.44	1.13	0.40
LaS_2	0.66	2.61	2.38	1.87	$Mg_{149}Cl$	0.12	2.23	2.19	3.91
$LaSe_2$	0.28	2.21	1.95	1.48	$Mg_{149}Cr$	0.02	0	0.53	0
$LaTe_2$	0.11	1.40	1.30	1.15	$Mg_{149}Fe$	0.08	0.20	0.76	0
Li_2O	4.99	7.80	7.93	8.07	$Mg_{149}Ga$	0.48	0.57	1.16	0
Li_2O_2	2.02	4.60	2.64	5.53	$Mg_{149}H$	0.04	0.78	1.11	0
Li_2S	3.54	5.03	5.51	5.42	$Mg_{149}Hg$	0.57	0.98	1.45	0.74
Li_2Se	3.11	4.53	5.03	4.80	$Mg_{149}I$	0.00	1.43	1.55	2.60
Li_2Te	2.52	3.20	3.94	4.00	$Mg_{149}In$	0.53	0.56	1.18	0
Li_3As	0.64	1.30	2.06	2.34	$Mg_{149}Mn$	0.11	0	0.50	0
Li_3Bi	0.38	0.76	1.62	1.12	$Mg_{149}Mo$	0.15	0.82	1.18	0
Li_3N	1.00	2.95	3.30	4.56	$Mg_{149}Nb$	0.12	0	0.56	0
Li_3P	0.92	1.61	2.36	2.83	$Mg_{149}Ni$	0.02	0.28	0.78	0
Li_3P_7	1.79	2.56	2.44	5.31	$Mg_{149}P$	0.00	0.72	1.06	1.62
Li_3Sb	0.81	1.27	2.10	1.98	$Mg_{149}Pb$	0.38	1.32	1.60	0.43
LiAs	0.36	0.99	1.76	2.35	$Mg_{149}Pd$	0.01	0.75	1.08	0
LiB_3	0.09	0.48	1.34	1.05	$Mg_{149}Pt$	0.02	0.88	1.17	0
LiBr	4.94	7.09	7.37	8.16	$Mg_{149}Re$	0.11	0.36	0.87	0
LiCl	5.93	8.43	8.62	9.58	$Mg_{149}Rh$	0.04	0.91	1.20	0
LiF	8.72	12.53	12.40	14.21	$Mg_{149}Ru$	0.07	0.80	1.13	0
LiH	3.18	4.07	4.73	16.20	$Mg_{149}S$	0.16	1.47	1.63	2.16
LiI	4.38	6.06	6.48	6.99	$Mg_{149}Sb$	0.27	0.76	1.19	1.10
LiN_3	3.66	5.83	6.12	6.27	$Mg_{149}Sc$	0.25	0	0.45	0
LiP	0.85	1.55	2.29	3.00	$Mg_{149}Se$	0.17	1.44	1.61	1.91
LiP_7	1.66	2.41	2.02	8.00	$Mg_{149}Si$	0.34	0.59	1.11	0.64
LiS_4	2.16	3.55	3.19	4.35	$Mg_{149}Sn$	0.42	0.76	1.25	0.43
Lu_2O_3	4.03	4.73	4.65	4.39	$Mg_{149}Ta$	0.15	0	0.49	0
Lu_2S_3	2.66	3.40	2.99	2.67	$Mg_{149}Tc$	0.10	0.34	0.85	0
Lu_2Se_3	1.36	2.44	2.04	1.85	$Mg_{149}Te$	0.15	0.73	1.12	1.58
$Mg_{149}Ti$	0.20	0	0.58	0	MoF_6	4.17	5.92	6.40	5.50
$Mg_{149}Tl$	0.49	0.97	1.41	0	MoI_2	1.68	2.02	2.41	1.64
$Mg_{149}V$	0.08	0	0.55	0	MoO_2	0.47	1.99	1.97	1.66
$Mg_{149}Zn$	0.58	0.37	1.09	0.50	MoO_3	1.65	3.04	3.20	2.23
$Mg_{149}Zr$	0.03	0	0.19	0	MoS_2	1.46	1.71	2.11	1.34
Mg_2Ge	0.25	0.68	1.13	0.73	$MoSe_2$	1.59	1.78	2.21	1.25
Mg_2Si	0.29	0.54	1.06	0.84	N_2O	6.24	5.53	7.08	7.63

Mg_3As_2	1.43	2.13	2.29	2.35	N_2O_5	0.69	1.09	1.27	4.09
Mg_3N_2	1.51	3.46	3.11	4.20	Na_2O	2.04	4.66	2.57	5.84
Mg_3P_2	1.61	2.33	2.12	2.72	Na_2O_2	1.77	4.37	2.85	5.42
Mg_3Sb_2	0.16	0.65	1.07	1.17	Na_2S	2.44	3.90	4.12	4.62
$MgAs_4$	0.84	1.54	1.92	2.61	Na_2S_5	1.80	3.21	2.10	4.01
MgB_4	0.37	0.84	1.28	1.45	Na_2Se	2.28	3.69	3.65	4.21
MgB_7	1.46	1.94	2.11	7.14	Na_2Te	2.28	3.00	3.75	3.86
$MgBr_2$	4.46	6.30	6.53	6.61	Na_3As	0.06	0.73	1.51	1.95
$MgCl_2$	5.67	7.78	8.01	7.97	Na_3As_7	1.42	2.20	1.90	4.61
MgF_2	6.83	9.99	10.08	10.90	Na_3P	0.40	1.12	1.87	2.49
MgH_2	3.90	4.63	5.15	12.31	Na_3P_{11}	1.97	2.80	3.05	3.20
MgI_2	3.68	5.10	5.39	5.44	Na_3Sb	0.39	0.88	1.72	1.71
MgO	4.64	7.11	7.22	7.30	$NaAs$	0.58	1.29	2.05	2.54
MgP_4	0.67	1.39	1.75	2.79	$NaBr$	4.09	6.22	6.54	7.53
MgS	2.96	4.27	4.57	4.65	$NaCl$	5.15	7.64	7.85	9.01
MgS_2	1.13	2.44	2.65	3.21	$NaCl_3$	2.71	5.01	4.36	6.68
$MgSe$	2.55	3.81	4.09	4.08	$NaCl_7$	0.52	2.65	3.01	6.25
$MgTe$	2.36	2.94	3.43	3.62	NaF	6.10	9.74	9.70	12.15
Mn_2O_3	0.08	2.67	2.19	2.67	$NaGe$	1.12	1.60	2.04	1.79
Mn_3O_4	0.84	3.33	2.99	2.98	NaH	4.07	5.09	5.72	19.87
Mn_4Si_7	0.80	1.20	1.34	0.40	NaI	3.57	5.24	5.68	6.38
$MnBr_2$	1.61	3.41	3.17	3.54	$NaMg_{149}$	0.55	0	1.10	0.22
$MnCl_2$	1.80	3.82	3.18	4.10	NaN_3	4.09	6.33	6.62	6.55
MnF_2	2.54	5.43	3.27	6.24	NaP	0.89	1.64	2.38	3.06
MnF_3	0.51	3.67	3.21	5.19	NaP_7	1.74	2.56	2.34	7.92
MnF_4	0.20	3.40	2.88	5.16	NaS	1.26	2.63	2.49	3.84
MnI_2	1.18	2.65	2.53	2.78	NaS_2	2.04	3.47	2.86	4.23
MnN	0.09	2.20	1.79	2.65	$NaSb$	0.65	1.15	1.98	2.10
MnO	0.20	2.77	2.32	2.91	$NaSe$	0.49	1.76	2.35	2.96
MnO_2	1.17	3.62	3.34	3.47	$NaSe_2$	1.21	2.53	2.51	3.41
MnP_4	0.52	1.41	1.35	2.05	$NaSi$	1.42	1.75	1.64	2.10
Mo_8O_{23}	0.91	2.38	2.43	0.98	$NaSn$	0.53	0.88	1.77	1.17
$MoBr_2$	2.12	2.83	3.19	2.17	$NaTe_3$	0.70	1.29	2.09	2.77
$MoCl_2$	2.47	3.40	3.12	2.61	Nb_2F_5	0.16	3.17	2.79	3.81
$MoCl_6$	1.08	2.17	2.31	3.16	Nb_2O_5	1.67	3.90	3.66	2.88
MoF_3	3.64	5.46	5.14	4.22	Nb_2Se_9	0.80	1.95	1.97	2.63
Nb_3Br_8	0.06	1.84	1.62	3.80	P_2O_5	5.20	5.74	6.89	5.77
Nb_3Cl_7	0.98	2.92	2.79	3.10	P_2Os	0.86	0.62	1.04	0.20
Nb_3Cl_8	0.15	2.18	1.92	4.26	P_2Pd	0.41	0.26	0.57	0
Nb_3I_8	0.08	1.45	1.33	3.38	P_2Pt	1.13	0.96	1.41	0.47
$NbBr_4$	0.87	2.57	2.47	2.60	P_2Rh	0.54	0.49	0.80	0
$NbBr_5$	1.56	3.19	3.19	3.13	P_2Ru	0.52	0.35	0.68	0
$NbCl_4$	1.08	3.01	2.90	3.06	P_2S_5	2.58	2.55	3.23	3.23
$NbCl_5$	2.22	4.04	2.79	3.79	P_2S_7	2.04	2.12	2.67	4.88

NbF_5	4.58	7.17	7.42	6.29	P_3Ir	0.09	0.01	0.24	0
NbI_4	0.49	1.82	1.75	1.99	P_3N_5	3.56	3.94	4.75	4.50
NbI_5	0.99	2.28	2.28	2.43	P_3Ru	1.03	0.76	1.22	0
NbO_2	0.30	2.66	2.37	2.30	P_4Os	1.30	0.98	1.51	0.17
NCl_3	2.16	1.92	2.52	5.38	P_4Ru	1.12	0.84	1.32	0
$Nd_{10}S_{19}$	0.50	1.80	1.79	1.33	P_4S_3	2.87	2.78	3.54	2.83
$Nd_{10}Se_{19}$	0.28	1.62	1.69	1.06	P_4S_7	2.78	2.71	3.45	4.19
Nd_2O_3	3.71	4.64	3.65	4.17	P_4S_9	2.72	2.66	3.39	3.96
Nd_2S_3	0.76	2.00	1.87	1.73	P_7Pb	0.89	0.84	1.21	0.28
Nd_2Se_3	1.43	2.50	1.43	1.85	$PaBr_4$	0.19	2.75	1.71	3.63
Nd_2Te_3	0.02	1.30	1.15	0.95	$PaBr_5$	1.96	4.12	1.21	4.86
$NdBr_3$	2.92	3.81	2.83	3.95	$PaCl_4$	0.16	2.96	1.91	4.14
$NdCl_3$	4.30	4.96	3.53	5.06	$PaCl_5$	2.42	4.71	0.96	5.65
NdF_3	7.81	8.07	5.44	8.43	Pb_3O_4	1.15	2.30	2.50	1.57
NdH_3	0.08	1.36	1.27	3.21	$PbBr_2$	2.73	2.97	3.18	2.53
NdI_3	2.02	3.00	0.99	3.00	$PbCl_2$	3.37	3.74	4.53	3.10
$NdMg_{149}$	0.19	1.47	0.37	0.65	$PbCl_4$	2.15	2.76	3.25	2.83
NdN	0.43	1.93	2.25	2.60	PbF_2	4.44	5.57	6.51	4.71
NdO_2	3.51	4.49	3.59	4.37	PbF_3	2.09	3.67	4.05	3.78
NdP_5	0.06	1.35	1.25	1.82	PbF_4	2.00	3.60	3.95	3.93
NdS_2	0.49	1.79	1.79	1.79	PbI_2	2.41	2.31	2.83	2.05
NF_2	1.90	2.71	3.10	7.22	PbO	1.54	2.61	2.91	2.28
Ni_3O_4	0.40	2.34	2.15	2.34	PbO_2	0.01	1.38	1.31	1.57
$NiBr_2$	0.48	1.81	1.73	2.54	PBr_3	3.35	3.67	4.44	4.80
NiF_2	4.91	6.73	7.44	6.85	PBr_5	1.64	2.30	2.65	4.35
NiO	2.41	4.02	3.21	3.92	PBr_7	1.24	1.98	2.24	6.05
NiS_2	0.19	1.07	1.03	1.58	PbS	0.99	1.06	1.43	1.24
NO_2	2.86	2.83	3.55	5.42	PbS_2	0.81	0.91	1.24	1.15
$NpCl_3$	0.01	2.42	2.02	4.55	$PbSe$	0.91	0.95	1.31	1.05
NpF_6	1.15	3.69	3.26	8.18	PCl_3	4.08	4.50	5.42	5.67
OF_2	2.13	2.54	2.92	7.46	PCl_5	1.79	2.68	3.03	4.91
OsF_5	0.72	2.79	2.75	3.73	$PdBr_2$	0.94	1.73	1.92	1.92
OsO_4	3.30	4.36	4.59	3.16	$PdCl_2$	1.43	2.39	2.64	2.45
$OsSe_2$	0.58	0.85	1.11	0.91	PdF_4	0.79	2.84	2.82	3.68
P_2Ir	0.78	0.56	0.96	0.17	PdI_2	0.96	1.33	1.62	1.56
PdS	0.01	0.43	0.54	0.81	PtI_4	0.69	0.99	1.26	1.67
PdS_2	0.65	0.97	1.22	1.19	PtO_2	0.66	1.99	2.04	1.89
$PdSe_2$	0.01	0.39	0.51	0.77	PtS	0.47	0.70	0.94	0.99
PF_5	7.11	7.87	9.45	9.60	PtS_2	1.44	1.51	1.96	1.46
PI_2	2.06	2.24	2.78	3.47	$PtSe_2$	0.67	0.82	1.12	0.99
PI3	2.36	2.48	2.74	3.70	PuF_3	0.00	2.48	2.96	6.99
Pm_2O_3	3.78	4.57	3.81	4.25	Rb_2As_3	0.64	1.49	2.23	1.74
Pm_2S_3	2.57	3.28	2.49	2.62	Rb_2O	0.48	3.09	3.38	3.61
Pm_2Se_3	0.50	1.68	1.79	1.44	Rb_2O_2	1.84	4.55	2.67	4.34

$PmBr_3$	2.89	3.67	2.86	3.98	Rb_2P_3	1.07	1.96	2.17	2.19
$PmCl_3$	3.54	4.26	3.44	4.73	Rb_2S	1.99	3.54	2.83	3.39
PmF_3	5.10	5.87	4.81	7.16	Rb_2S_3	1.36	2.85	2.27	2.77
PmI_3	2.07	2.93	1.19	3.07	Rb_2S_5	1.98	3.52	2.78	3.22
$PmMg_{149}$	0.20	1.37	0.39	0.63	Rb_2Se	1.82	3.31	2.31	3.03
PmS_2	0.46	1.67	1.80	1.80	Rb_2Se_3	0.69	2.09	2.67	2.18
$Pr_{10}S_{19}$	0.51	1.83	1.79	1.25	Rb_2Se_5	0.84	2.25	2.78	2.37
$Pr_{10}Se_{19}$	0.31	1.67	1.70	0.99	Rb_2Te	1.89	2.72	3.03	2.79
$Pr_{13}Zn_{58}$	0.16	1.38	0.72	0	Rb_2Te_3	0.74	1.47	2.24	1.91
Pr_2Br_5	0.29	1.81	2.12	2.43	Rb_2Te_5	0.40	1.10	1.89	1.85
Pr_2I_5	0.13	1.57	1.77	1.87	Rb_3As_{11}	1.47	2.38	1.73	1.98
Pr_2O_3	3.66	4.64	3.51	4.00	Rb_3As_7	1.46	2.37	1.75	3.81
Pr_2S_3	0.78	2.04	1.85	1.65	Rb_3P_{11}	1.93	2.90	3.05	2.40
Pr_2Se_3	1.43	2.53	1.37	1.76	Rb_3Sb	0.09	0.69	1.51	0.96
Pr_2Te_3	0.88	1.98	1.38	1.27	Rb_4Pb_9	0.30	1.35	2.03	0.46
Pr_4Ge_3	0.15	1.40	1.09	0	$RbAs$	1.05	1.92	2.18	2.20
$PrBr_3$	3.43	4.24	2.95	4.06	$RbAu$	0.72	2.11	2.69	0.57
$PrCl_3$	4.35	5.04	3.40	4.91	$RbBi$	0.38	0.95	1.79	0.89
PrF_3	7.60	7.97	5.11	8.08	$RbBr$	4.37	6.64	6.95	6.42
PrH_3	0.29	1.54	1.33	3.47	$RbCl$	5.02	7.60	7.83	7.40
PrI_3	1.96	2.98	0.77	2.85	RbF	5.65	9.36	9.36	9.83
$PrMg_{149}$	0.18	1.46	0.37	0.63	RbF_3	2.11	5.54	2.94	7.00
PrN	0.41	1.94	2.24	2.48	$RbGa_3$	0.62	0.87	1.82	0.27
PrP_5	0.04	1.35	1.25	1.73	$RbGe$	0.92	1.52	2.14	1.16
PrS_2	0.49	1.82	1.78	1.70	RbH	3.20	4.28	4.93	15.62
$PrSe_2$	0.00	1.43	1.62	1.26	RbI	3.95	5.77	6.20	5.51
PSe	2.25	2.24	2.74	3.10	RbI_3	1.96	3.62	2.66	3.84
Pt_3I_8	0.80	1.08	1.37	4.24	$RbMg_{149}$	0.02	0	0.67	0
$PtBr_3$	0.94	1.61	1.83	1.93	RbN_3	4.20	6.56	6.85	5.36
$PtCl_2$	1.83	2.61	2.97	2.52	RbP_{15}	1.51	2.44	1.71	2.24
$PtCl_3$	1.07	1.98	2.17	2.29	$RbPb$	0.41	1.46	2.14	0.68
PtF_4	1.29	3.15	3.27	3.74	RbS	1.58	3.09	2.04	3.23
PtI_2	1.25	1.46	1.84	1.60	$RbSb$	0.69	1.34	2.14	1.55
PtI_3	0.42	0.77	0.97	1.37	$RbSb_2$	0.31	0.93	1.75	1.31
$RbSe$	0.92	2.34	2.69	2.53	SbF_3	4.54	6.05	6.91	6.57
$RbSi$	1.35	1.81	1.57	1.52	SbF_4	3.44	5.17	4.63	6.18
$RbSn$	0.74	1.25	2.11	0.88	SbF_5	3.68	5.36	5.38	6.59
$RbTe$	0.61	1.33	2.11	2.02	SbI_3	2.21	2.61	2.75	2.98
Re_2O_7	2.68	4.29	2.94	4.67	SbO_2	1.90	3.34	3.58	3.44
Re_2P_5	0.49	0.68	0.95	1.06	SBr	2.31	2.26	2.95	3.97
Re_2Te_5	0.61	0.64	0.98	0.87	Sc_2Ni	0.01	0.45	0.72	0
Re_3Br_7	0.36	1.67	1.62	2.06	Sc_2O_3	3.81	6.25	6.30	4.39
Re_4Si_7	0.03	0	0.16	0	Sc_2Pd	0.01	0.92	1.03	0
$ReCl_3$	0.97	2.46	2.47	2.46	Sc_2S_3	1.07	2.48	2.53	1.98

ReF_7	2.00	4.34	4.20	6.30	Sc_2Se_3	0.66	2.05	2.07	1.57
ReI_3	0.96	1.78	1.93	1.73	Sc_2Te_3	0.04	0.78	0.95	1.03
ReP_4	0.76	0.92	1.23	1.51	Sc_4C_3	0.47	1.87	1.87	1.08
ReS_2	1.45	2.10	2.36	1.59	$Sc_{57}Pt_{13}$	0.10	1.12	1.20	0
$ReSe_2$	1.24	1.87	2.11	1.35	$Sc_{57}Ru_{13}$	0.02	0.92	1.03	0
Rh_2S_3	0.19	0.45	0.65	0.60	$ScBr_3$	2.25	4.13	2.68	3.80
$RhBr_3$	1.31	1.93	2.22	2.08	$ScCl_3$	3.88	5.95	6.08	5.07
$RhCl_3$	1.56	2.40	2.69	2.48	ScF_3	6.11	9.10	9.27	7.90
RhF_3	0.89	2.83	2.85	3.40	ScI_3	1.70	3.20	2.56	3.00
RhF_6	0.88	2.82	2.84	4.48	SCl	2.89	2.98	3.20	4.80
RhI_3	1.06	1.30	1.64	1.62	SCl_4	2.62	2.76	3.40	4.67
RuF_4	0.19	2.34	2.19	3.22	ScN	0.33	2.40	2.23	2.69
RuO_4	2.43	3.61	3.50	2.84	Se_2O_5	3.02	3.47	3.36	3.93
RuS_2	0.68	0.98	1.25	1.09	$SeBr_4$	2.21	2.22	2.88	3.70
$RuSe_2$	0.48	0.76	1.00	0.87	$SeCl_4$	3.04	3.14	3.70	4.51
S_7N	0.65	1.05	1.29	2.23	SeF_4	4.14	4.98	5.97	6.61
S_8O	2.42	2.95	3.57	3.55	SeO_2	3.36	3.73	4.40	4.20
Sb_2F_7	3.83	5.48	5.85	7.19	SeS_7	1.90	1.46	2.15	7.31
Sb_2Ir	0.45	0.55	0.76	0	SF_4	4.88	5.53	6.71	7.51
Sb_2O_3	2.23	3.60	3.54	3.22	SF_6	5.91	6.35	7.79	9.17
Sb_2O_5	0.79	2.45	2.42	2.86	Si_2Os	0.65	1.00	1.13	0
Sb_2Os	0.40	0.52	0.71	0	Si_2Ru	0.48	0.86	0.95	0
Sb_2Rh	0.00	0.32	0.38	0	Si_3N_4	4.26	5.03	5.78	4.20
Sb_2Ru	0.09	0.26	0.38	0	Si_3Os_2	0.86	1.17	1.34	0
Sb_2S_3	1.31	1.78	2.06	1.78	Si_3Ru_2	0.55	0.92	1.02	0
Sb_2Se_3	0.86	1.38	1.55	1.38	Si_5Ir_3	0.93	1.23	1.43	0
Sb_2Te_3	0.16	0.17	0.35	0.83	Si_7Tc_4	1.07	0.86	1.25	0.89
Sb_3Ir	0.53	0.61	0.84	0	SiAs	1.48	1.64	1.98	2.05
Sb_3Rh	0.23	0.49	0.61	0	$SiAs_2$	0.90	1.17	1.36	1.67
Sb_7F_{29}	3.60	5.30	5.11	4.94	SiB_3	1.49	1.43	1.84	1.38
$SbBr_3$	3.45	3.99	4.70	4.07	$SiBr_4$	4.11	4.81	5.54	4.89
$SbCl_3$	3.75	4.48	5.22	4.62	SiC	2.04	2.62	2.65	2.51
$SbCl_5$	1.84	2.95	3.22	4.14	$SiCl_4$	5.79	6.41	7.51	6.17
SiF_4	7.94	9.08	10.62	8.95	SO_3	5.14	5.11	6.42	5.48
SiH_8	7.35	6.39	8.14	10.39	Sr_2Ge	0.37	1.46	1.64	0.72
SiI_3	2.96	3.50	4.02	3.66	Sr_2Pb	0.04	1.61	1.62	0.34
SiI_4	2.86	3.42	3.92	3.74	Sr_2Sb_3	0.13	1.28	1.42	1.16
SiO_2	5.85	6.79	7.87	5.81	Sr_2Si	0.34	1.26	1.49	0.77
SiP	1.82	1.92	2.34	2.46	Sr_2Sn	0.15	1.16	1.35	0.39
SiP_2	1.49	1.66	1.99	2.17	Sr_3As_4	0.68	2.03	2.13	1.83
SiRu	0.25	0.68	0.71	0.18	Sr_3P_{14}	1.82	3.18	2.57	2.33
SiS_2	3.14	3.54	4.13	3.31	Sr_3P_2	0.14	1.50	1.58	1.73
$SiSe_2$	2.18	2.73	2.51	2.60	Sr_3P_4	0.89	2.25	2.36	2.18
Sm_2O_3	3.88	4.74	3.92	4.11	Sr_4As_3	0.59	1.93	2.03	1.70

Sm_2S_3	0.74	1.99	1.88	1.64	$SrAs$	0.02	1.37	1.44	1.61
Sm_2Se_3	0.51	1.80	1.76	1.34	SrB_6	0.04	1.17	1.31	4.26
Sm_2Te_3	0.92	2.00	1.44	1.29	$SrBr_2$	4.47	6.90	6.92	6.21
$SmBr_3$	2.93	3.80	3.01	3.83	$SrCl_2$	5.23	7.92	7.92	7.21
$SmCl_3$	3.59	4.39	3.52	4.56	SrF_2	6.78	10.48	10.40	10.21
SmF_3	7.86	8.05	5.94	8.24	$SrGe_2$	0.40	1.49	1.66	0.92
SmH_3	0.13	1.42	1.26	3.42	SrH_2	3.24	4.61	4.83	9.31
SmI_3	2.12	3.07	1.33	2.94	SrI_2	3.80	5.83	5.90	5.17
$SmMg_{149}$	0.21	1.51	0.36	0.71	SrN_6	3.67	6.20	6.16	6.53
SmN	0.02	1.62	2.10	2.25	SrO	3.45	6.49	6.35	6.03
SmP_5	0.06	1.37	1.22	1.77	SrO_2	2.91	5.95	4.66	5.31
SmS_2	0.54	1.84	1.81	1.73	SrP	0.75	2.11	2.21	2.36
Sn_3F_8	3.22	5.17	4.03	8.80	SrP_2	0.81	2.17	2.28	2.35
Sn_3N_4	0.47	1.87	1.76	1.93	SrP_3	0.38	1.74	1.83	2.18
Sn_5O_6	1.77	3.39	3.53	2.76	SrS	2.56	4.48	4.48	4.07
$SnBr_2$	2.20	3.16	2.98	3.08	SrS_3	1.29	3.21	2.48	3.07
$SnBr_4$	2.71	3.57	3.50	3.50	$SrSe$	2.29	4.17	3.67	3.63
$SnCl_2$	3.11	4.14	4.53	3.91	$SrTe$	2.04	3.27	3.36	3.16
$SnCl_4$	3.69	4.60	5.25	4.31	Ta_2O_5	2.29	4.64	2.91	3.41
SnF_2	3.27	5.21	4.20	5.44	Ta_3Br_7	1.27	3.12	2.99	3.11
SnF_3	2.92	4.93	3.29	5.28	Ta_3I_7	0.89	2.37	2.28	2.43
SnF_4	3.20	5.15	3.97	5.55	Ta_3N_5	1.26	3.21	3.06	2.62
SnI_2	1.83	2.48	2.79	2.46	$TaBr_5$	2.03	3.81	2.67	3.58
SnI_4	2.03	2.63	2.85	2.75	$TaCl_5$	2.82	4.77	4.12	4.34
SnO	0.63	2.48	2.34	2.49	TaF_5	5.56	8.22	8.54	7.16
SnO_2	1.41	3.10	3.16	2.87	TaI_5	1.17	2.63	2.58	2.70
SnS	1.11	1.79	1.95	1.83	Tb_2Cl_3	0.87	2.27	2.57	3.06
SnS_2	1.56	2.15	2.42	2.00	Tb_2O_3	3.91	4.69	4.21	4.20
$SnSe$	0.82	1.51	1.61	1.49	Tb_2S_3	0.58	1.84	1.83	1.60
$SnSe_2$	0.80	1.50	1.59	1.47	Tb_2Se_3	0.55	1.80	1.79	1.39
$SnTe$	0.66	0.73	0.97	1.18	Tb_2Te_3	0.79	1.88	1.43	1.27
SO_2	3.20	3.57	3.89	4.47	Tb_8Se_{15}	0.32	1.63	1.69	1.03
$TbBr_3$	2.86	3.69	3.03	3.86	$ThBr_4$	2.95	4.58	2.49	5.36
$TbCl_3$	4.65	5.11	4.24	5.16	$ThCl_4$	3.75	5.30	2.91	6.37
TbF_3	7.95	7.99	6.56	8.40	ThF_4	6.54	7.87	4.38	10.06
TbH_3	0.00	1.30	1.18	3.37	ThI_4	1.85	3.61	0.82	4.09
TbI_3	2.10	3.00	1.41	2.99	$ThMg_{149}$	0.02	0.86	0.15	1.00
$TbMg_{149}$	0.25	1.54	0.36	0.78	ThO_2	4.46	5.98	3.36	6.31
TbN	0.19	1.71	2.14	2.41	ThS_2	1.03	2.95	1.70	2.94
TbS_2	0.48	1.76	1.79	1.75	$ThSe_2$	0.65	2.64	1.58	2.46
Tc_2O_7	2.67	4.29	2.94	4.67	$ThSe_3$	1.50	3.30	1.36	3.01
Tc_2Te_5	0.53	0.58	0.89	0.83	$ThTe_3$	1.09	2.87	1.21	2.47
$TcBr_3$	0.08	1.43	1.33	1.75	Ti_2Sn	0.05	0.38	0.62	0
$TcCl_3$	0.68	2.20	2.16	2.33	$TiBr_3$	0.02	1.82	1.64	2.37

$TcCl_4$	0.78	2.30	2.27	2.53	$TiBr_4$	2.84	4.43	4.35	3.85
TcF_4	1.30	3.74	3.68	4.02	$TiCl_3$	0.03	2.10	1.86	2.79
TcP_3	0.39	0.60	0.84	1.11	$TiCl_4$	3.47	5.28	5.46	4.55
TcP_4	0.85	1.00	1.32	1.55	TiF_4	4.17	6.93	7.05	6.45
TcS_2	1.18	1.87	2.08	1.48	TiI_3	0.20	1.58	1.52	1.98
Te_2Br	0.77	1.77	1.84	2.53	TiI_4	1.11	2.42	2.47	2.55
Te_2I	0.66	1.29	1.41	1.96	TiO_2	2.69	4.91	3.38	3.79
Te_2Mo	1.86	1.53	2.14	1.61	TiS_3	0.23	1.50	1.47	1.67
Te_2O_5	1.70	3.08	3.31	3.46	Tl_2Cl_3	1.77	2.92	3.16	1.99
Te_2Os	0.52	0.53	0.79	0	Tl_2O	0.57	2.28	2.20	1.36
Te_2Ru	0.32	0.37	0.57	0	Tl_2O_3	0.03	1.84	1.63	1.19
Te_3As_2	0.58	0.54	0.83	0.91	Tl_2S	0.90	1.46	1.64	0.80
Te_4Mo_3	0.01	0.06	0.21	0	Tl_2S_5	1.70	2.11	2.48	1.37
$TeBr_4$	2.52	3.16	2.79	3.97	Tl_2Te	0.65	0.55	0.88	0.37
$TeCl_4$	3.11	3.87	4.39	4.68	Tl_4O_3	1.20	2.79	2.85	1.40
TeF_4	4.02	5.53	6.31	6.77	$TlBr$	2.35	3.14	2.91	2.48
TeF_6	4.40	5.83	6.70	7.88	$TlBr_2$	1.81	2.70	2.99	2.05
TeI	0.73	1.35	1.49	2.31	$TlCl$	2.60	3.59	2.86	2.91
TeI_4	1.76	2.17	2.57	3.10	$TlCl_2$	2.31	3.36	3.15	2.56
TeO_2	2.24	3.51	3.59	3.80	$TlCl_3$	2.35	3.38	3.12	2.61
TeO_3	2.16	3.45	3.67	3.83	TlF	3.26	5.08	4.08	4.40
$TePb$	1.06	1.14	1.48	0.88	TlF_3	1.27	3.46	3.49	3.33
$TeSe$	0.95	1.37	1.60	1.89	TlI	2.44	2.81	2.86	2.18
$TeSe_2$	0.90	1.33	1.54	1.86	TlI_3	1.85	2.34	2.72	1.79
Th_2S_5	2.12	3.79	0.97	3.51	TlP_5	1.42	1.32	1.78	2.10
Th_2Se_5	1.34	3.18	1.66	2.82	$TlSe$	0.36	0.98	1.04	0.69
Th_3As_4	0.28	2.26	1.12	1.62	$Tm_{10}S_{19}$	0.53	1.83	1.80	1.27
Th_3Bi_4	0.21	2.18	0.93	0.68	Tm_2O_3	3.99	4.72	4.51	4.23
Th_3N_4	1.67	3.62	1.55	4.05	Tm_2S_3	2.27	3.12	2.07	2.39
Th_3P_4	0.27	2.25	1.12	1.86	Tm_2Se_3	1.37	2.44	1.96	1.77
Th_3Sb_4	0.17	2.15	0.96	1.14	Tm_2Te_3	0.58	1.76	1.33	1.17
$TmBr_3$	2.99	3.78	3.49	3.93	XeF_6	2.79	3.80	4.11	6.92
$TmCl_3$	5.02	5.36	4.76	5.35	Y_2Br_3	0.70	2.91	2.69	2.84
TmF_3	5.51	6.10	5.87	7.20	Y_2Cl_3	0.86	3.32	3.06	3.37
TmH_3	0.06	1.39	1.16	3.67	Y_2O_3	4.11	6.77	6.75	4.65
TmI_3	2.27	3.14	1.98	3.08	Y_2S_3	1.60	3.26	2.44	2.31
TmN	0.31	1.83	2.17	2.49	Y_2Se_3	1.35	2.98	2.66	1.99
$U_{11}Se_{20}$	0.00	2.31	1.36	1.94	Y_2Te_3	0.79	1.79	1.89	1.46
U_3O_8	0.03	2.68	2.30	3.67	Y_4C_7	0.61	2.27	2.17	1.31
UBr_4	0.22	2.62	1.85	4.23	$Yb_{13}Zn_{58}$	0.07	0.64	0.75	0
UBr_5	0.29	2.67	1.87	4.47	Yb_2Ge	0.04	0.63	1.11	0.26
UCl_5	0.33	2.79	2.10	5.08	Yb_2Si	0.04	0.62	0.99	0.33
UCl_6	1.58	3.75	2.05	6.25	Yb_3N_2	0.49	1.27	2.43	2.84
UF_3	0.22	3.09	2.92	6.96	YbB_6	0.11	0.68	1.17	4.74

UF_5	0.75	3.50	3.08	7.60	$YbBr_2$	4.88	4.47	4.71	5.73
UF_6	2.80	5.08	1.22	9.30	$YbCl_2$	5.40	4.93	5.21	6.50
UI_4	0.26	2.54	1.55	3.57	YbF_2	6.73	6.31	6.78	9.11
UN_2	0.77	3.08	2.10	4.34	YbH_2	2.89	2.76	2.84	9.20
UO_3	1.65	3.92	2.22	5.22	YbI_2	4.01	3.72	3.93	4.67
V_2O_3	0.49	2.78	2.54	2.44	$YbMg_{149}$	0.49	1.02	0.62	0.57
V_2O_5	2.34	4.45	2.99	3.52	YbO	3.53	3.68	4.49	5.42
V_2Se_9	0.77	1.86	1.90	2.85	YBr_3	4.05	6.11	6.20	4.82
V_3O_5	1.15	3.37	3.23	2.78	YbS	2.44	2.54	2.72	3.50
VBr_2	1.19	2.80	2.77	2.86	YbS_2	1.64	1.95	1.43	2.80
VCl_2	1.26	3.12	3.05	3.31	YbSe	2.03	2.22	1.67	3.00
VCl_5	1.05	2.93	2.83	3.62	YbTe	1.53	1.74	1.11	2.39
VF_3	1.64	4.46	4.29	5.10	YCl_3	4.98	7.25	7.37	5.74
VF_4	2.31	5.07	3.58	5.55	YF_3	7.65	10.81	11.03	8.85
VF_5	2.99	5.68	3.66	6.10	YH_3	0.01	1.19	1.17	3.46
VI_2	1.11	2.32	2.37	2.35	YI_3	3.06	4.76	4.84	3.78
VS_4	0.95	2.07	2.13	2.16	YMg_{149}	0.27	0	0.51	0.83
WBr_2	2.47	2.88	3.12	2.08	YN	0.29	2.62	2.34	2.74
WBr_6	1.13	1.70	1.95	2.78	Zn_3As_2	0.14	1.07	0.83	1.21
WCl_2	2.83	3.47	3.24	2.49	Zn_3N_2	0.43	2.45	2.04	2.99
WCl_4	0.28	1.22	1.26	1.81	Zn_3P_2	0.61	1.47	1.33	1.70
WCl_6	1.92	2.67	2.98	3.34	Zn_6Ru	0.01	0.99	0.72	0
WF_6	5.36	6.75	7.44	5.66	$ZnAs_2$	0.22	1.14	0.92	1.69
WI_2	1.96	2.01	2.50	1.56	$ZnBr_2$	3.22	4.63	4.88	5.10
WO_3	1.15	2.35	2.47	1.86	$ZnCl_2$	4.06	5.56	5.96	6.11
WS_2	1.62	1.59	2.06	1.24	ZnF_2	3.48	6.02	5.16	7.71
WSe_2	1.51	1.45	1.91	1.08	ZnI_2	2.91	4.00	4.23	4.31
XeF_2	2.82	3.83	4.06	5.62	ZnO	0.73	3.17	2.77	3.81
XeF_3	2.64	3.69	4.25	5.59	ZnP_2	1.47	2.17	2.23	2.60
XeF_4	2.86	3.87	4.02	5.89	ZnS	1.21	2.51	2.38	2.88
ZnS_2	2.00	3.15	2.46	3.26	ZrI_2	0.30	2.08	1.84	2.13
ZnSb	0.08	0.82	0.63	1.12	ZrI_3	0.20	1.98	1.73	2.15
ZnSe	1.61	2.79	2.77	2.92	ZrI_4	2.01	3.66	2.28	3.15
ZnTe	1.08	1.72	1.73	2.26	ZrO_2	3.54	6.08	6.05	4.46
Zr_3N_4	1.27	3.48	3.26	2.79	ZrS_2	1.04	2.66	2.53	2.12
$ZrBr_4$	3.78	5.70	5.80	4.53	ZrS_3	1.09	2.70	2.59	2.20
$ZrCl_2$	0.86	3.25	2.95	3.36	$ZrSe_2$	0.34	1.97	1.77	1.56
$ZrCl_3$	0.54	2.95	2.61	3.25	$ZrSe_3$	0.56	2.17	1.99	1.75
$ZrCl_4$	3.62	5.81	5.84	4.88	$ZrTe_5$	0.08	1.06	1.02	1.66
ZrF_4	5.35	8.39	8.51	7.38	ZrZn	0.02	0.26	0.49	0.22
